# Tedium vitae, death wishes, suicidal ideation and attempts in Kenya-prevalence and risk factors

**DOI:** 10.1186/s12889-015-2089-3

**Published:** 2015-08-08

**Authors:** Rachel Jenkins, Caleb Othieno, Ray Omollo, Linnet Ongeri, Peter Sifuna, Michael Ongecha, James Kingora Mboroki, David Kiima, Bernhards Ogutu

**Affiliations:** Institute of Psychiatry, Kings College London, London, UK; University of Nairobi, Nairobi, Kenya; Centre for Clinical Research, Kenya Medical Research Institute, Nairobi, Kenya; Kombewa Health and Demographic Surveillance System, Kombewa, Kenya; Centre for Global Health, Kenya Medical Research Institute, Kisumu, Kenya; Kenya Medical Training Centre, Nairobi, Kenya; Kenya Ministry of Medical Services, Nairobi, Kenya

## Abstract

**Background:**

There has been no previous household population study of suicidal ideation and attempts in Kenya. Therefore this study aimed to establish the prevalence of suicidal ideation and attempts in a rural population in Kenya, and to assess risk factors.

**Methods:**

An epidemiological survey of a household population, using standardised structured interviews. We examined the prevalence of suicidal ideation and suicide attempts and the predictors of suicidal thoughts and attempts, using STATA to calculate unadjusted and adjusted odds ratios.

**Results:**

A quarter of the sample (24.1 %) had thought that life was not worth living (tedium vitae) at some point in their lives, while a fifth had experienced death wishes at some stage. About 7.9 % reported suicidal thoughts and 1.9 % had made actual suicide attempts at some point in their lives. It can be seen that the prevalence of suicidal thoughts was 0.7 %, 4.2 %, 3.7 % and 7.9 % for last week, last year, at some other time, and lifetime respectively, while the prevalence of suicidal attempts was 0.5 %, 1.2 %, 0.7 and 1.9 % respectively.

In the adjusted analysis of factors associated with suicidal thoughts, being female (OR 1.8, p = 0.017), having CMD (OR 2.7, p = 0.001), having a number of recent life events (OR 2.3, p = 0.001 for 2–3 life events and OR 2.6, p = 0.004 for 4 or more life events), and having a large social group size (OR 7.7, p = 0.006 for social group size of 4–8 and OR 9.1, p = 0.003 for social group size of 9 or more) were all associated with increased rates of life time suicidal thoughts, but psychotic symptoms were no longer significant after adjustment for the other variables. In the adjusted analysis of suicide attempts, having any psychotic symptoms (OR 5.1, p = 0.001) was the only factor associated with suicide attempts after adjustment for other factors significant at the bivariate level.

**Conclusion:**

Suicidal ideation and attempts pose a significant public health burden in this poor rural area of Kenya. The findings are relevant for mental health promotion and prevention programmes, public education and professional training programmes in relevant sectors, especially in front line health workers and social workers.

## Background

Suicide is a major cause of mortality across the world [1] and, while it was previously considered rare in Africa [2], it is likely that this arose from serious under-reporting rather than actual low rates. A study of mortality in women aged 15–59 in Tanzania [3], in a demographic surveillance site in which censuses were carried out annually to determine denominator populations, found a suicide rate similar to English rates. Indeed WHO has recently pointed out that suicide is a global public health problem because, numerically, most suicides occur in low- and middle-income countries [4]. In seeking to understand the antecedents of actual suicide, it is useful not only to study actual suicides and their antecedents, but also to study suicidal ideation and suicide attempts, as these are important components of the population pathway to suicide [5]. Furthermore, suicide itself is relatively rare, whereas suicidal ideation and attempts are much more common, and are therefore less costly to obtain an adequate sample size for detailed study [6].

Models of the pathway to suicide proposed a continuum from depressive thoughts, to feeling that life is not worth living (tedium vitae), to feeling one would rather be dead (death wish), to suicidal thoughts to suicidal plans, to suicide attempts, and then to completed suicide [5]. Some studies of such a pathway have focused on suicidal thoughts, suicidal plans and suicidal attempts [6–8] and a few have included death wish as well as suicidal thoughts and attempts [9–13]. Baca Garcia has found that death wish is as predictive of suicidal attempts as is suicidal ideation, and that the best predictor is a combination of both [13], while Bebbington et al. found that all three (tedium vitae, death wish and suicidal thoughts) were independently predictive of suicidal attempts [12].

Population based epidemiological surveys are an essential tool for estimating population health, morbidity, co-morbidities, disability, associated risk factors and the extent to which health needs are met by the health services. All this information is needed to inform policy and planning [14] for meeting mental health needs in the general population and in vulnerable groups.

The study aimed to examine the prevalence and associated risk factors of tedium vitae, death wishes, suicidal ideation and attempts in a Kenyan household population.

## Methods

The opportunity was taken to examine prevalence and associated risk factors for tedium vitae, death wishes, suicidal ideation and attempts as part of a wider project to examine the associations between mental disorders, malaria and immunity in Maseno division of Kisumu County, near Lake Victoria in Kenya. The research was conducted as part of an overall collaborative programme of work between the Kenya Ministry of Health and the UK Institute of Psychiatry, Kings College London [15–29]. The study relies on data drawn from a community study of the prevalence of mental disorders, and their risk factors in the general population in Kisumu County, near Lake Victoria in Kenya. This repeat epidemiological survey is important to indicate the sustained mental health needs in the general adult population of Kenya, in a region of Kenya which experienced serious election violence in 2007/8 [30]. The data for this study was collected between December 2012 and June 2013.

### Study population

**Fig. 1:**
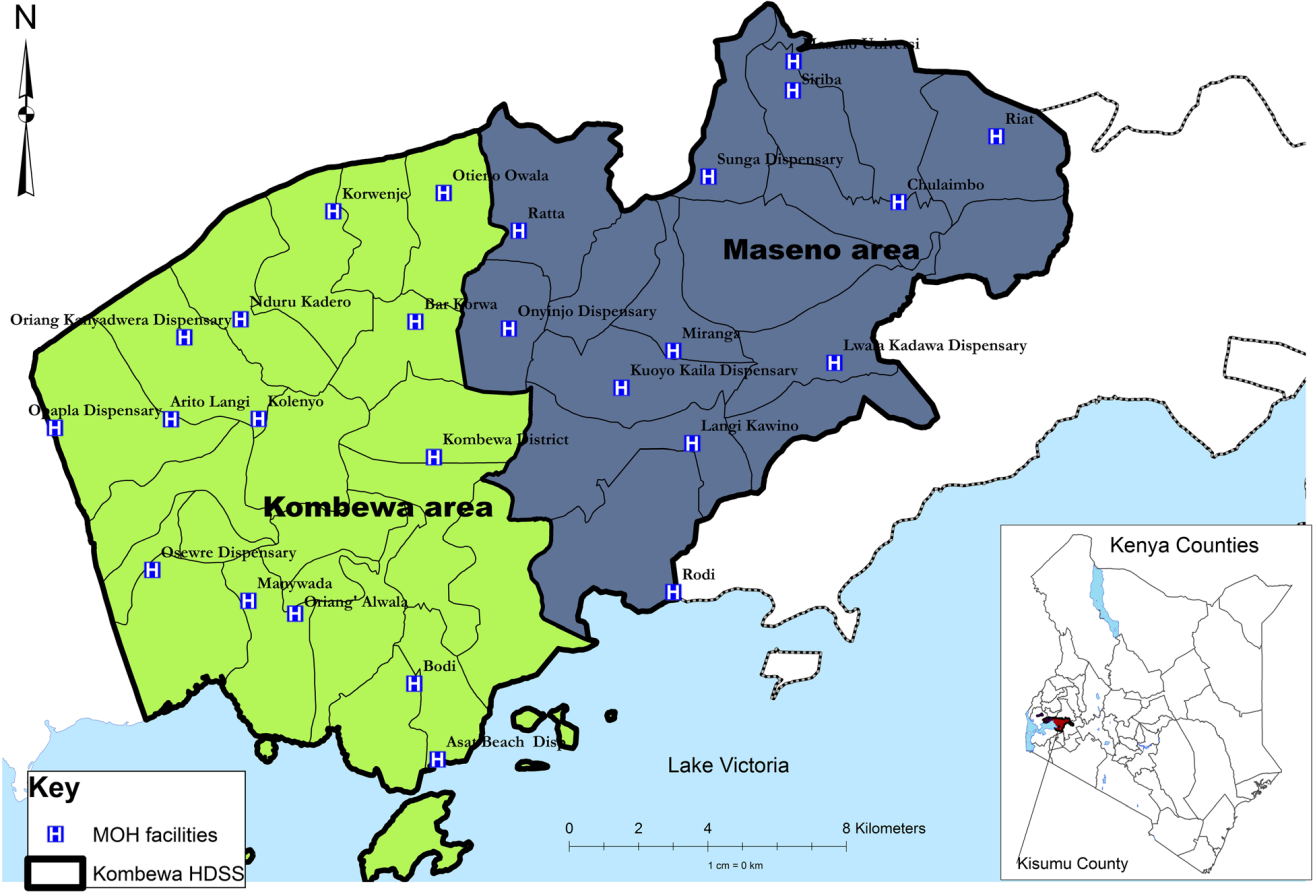
Study site

The sample frame is Maseno division of Kisumu County, in western Kenya (see Fig. 1). Maseno division has a population of 70,805 [31]. Females constitute 53 % of the population. The mean household size is 4 people per household. The area has a mean population density of 374 people/km^2^. The population is largely young with a mean age of 23 years. Just under half of the population (46 %) are aged 0-14years, nearly half (49 %) are aged 15–64, and the remaining 5 % are aged 65 and over.

### Study participants

The study sample was selected from Maseno division within Kisumu County, western Kenya. Maseno division is sub-divided into 4 locations, 17 sub-locations and 184 enumeration areas (villages) based on mapping work done earlier by the Kombewa Health and Demographic Surveillance System (Kombewa HDSS) run by the KEMRI/Walter Reed Project. The Kombewa HDSS is a longitudinal population registration system set up to monitor the evolving health and demographic problems of the study population in Kombewa and Maseno [31]. Some villages with less than 50 households were merged together to create new enumeration areas. A random sample of 7 households was drawn from each enumeration area. Village maps were used to assign households and guide the research assistants during the survey. Using the Kish Grid Method, one individual was selected from each of the sampled household. The demographics and reasons for the refusal were recorded in notebooks by the Research assistants.

### Study procedures

Meetings were held with community leaders to explain the purpose of the survey, and answer questions. The heads of the sampled households, and then the identified participants in the survey were approached in their own homes for informed written and witnessed consent to the interview. The interview was administered by one of a group of 20 research assistants using a PDA, on which the interview questions were programmed and responses were recorded. The research assistants received a 5 day training course, and were supervised in the field by a field manager.

The participants received a structured epidemiological assessment of common mental disorders, psychotic symptoms, alcohol and substance abuse, suicidal thoughts and attempts, accompanied by additional sections on socio-demographic data, life events, social networks, social supports, disability/activities of daily living, quality of life, use of health services, and service use, all of which sections were adapted from the Adult Psychiatric Morbidity Schedule [32] used in the British Psychiatric Morbidity Survey Programme.

Demographic information collected included age, sex, ethnicity, marital status and household status (head, spouse or other). Socio-economic factors assessed included employment status, education attainment, economic assets and type of housing.

Suicidal ideation was assessed by the following questions:

- Have you ever felt that life was not worth living? (Tiredness of life, tedium vitae). Was this in the last week/in the last year/or at some other time?
- Have you ever wished that you were dead? (Death wish). Was this in the last week/in the last year/or at some other time?
- Have you ever thought of taking your life, even if you would not really do it? (Suicidal thinking). Was this in the last week/in the last year/or at some other time?

Suicide attempts were assessed by the following:

- Have you ever made an attempt to take your life, by taking an overdose of tablets or in some other way? (Suicide attempt). Was this in the last week/in the last year/or at some other time?
- Have you deliberately harmed yourself in any way but not with the intention of killing yourself? (Self harm).

(The timing of acts of self-harm was not elicited, and the question about attempted suicide was only asked of participants who acknowledged having had suicidal ideation).

Common mental disorders (CMD) were assessed by the Clinical Interview Schedule-Revised (CIS-R) [33], a gold standard instrument for use by lay interviewers to assess psychopathology in community settings. It has been widely used in high- [34–36] and low-income countries [37–39], including Tanzania [40, 41] and Kenya [17]. The CIS-R measures the presence of 14 symptom-types in the preceding month and the frequency, duration and severity of each symptom in the past week. Scores, taken together with algorithms based on the ICD-10 [42], provide diagnoses of depressive episode (mild, moderate or severe), obsessive compulsive disorder, panic disorder, phobic disorder, generalised anxiety disorder and mixed anxiety/depressive disorder. For the purpose of the current paper however, a score of 12 or more across the 14 sections of the survey was considered an indication of “any CMD”, as used in other CIS-R studies [34–36].

Psychotic symptoms were assessed by the Psychosis Screening Questionnaire (PSQ) [43], which has also been used previously in Tanzania [44] and Kenya [18]. The PSQ assessed the past year presence of psychotic symptoms. The instrument developed for use by lay interviewers employs five probes to determine recent experience of mania, thought insertion, paranoia, strange experiences and hallucinations.

Questions were asked about current and life time use of alcohol, tobacco and drugs. The Alcohol Use Disorders Identification Test (AUDIT) [45] measured hazardous alcohol use.

Respondents were given a list of 18 different stressful life events and asked to say which, if any, they had experienced in the last six months. The list included:

- Serious illness, injury or assault to self;
- Serious illness, injury or assault to a close relative;
- Death of an immediate family member of yours;
- Death of a close family friend or other relative;
- Separation due to marital differences, divorce or steady relationship broken;
- Serious problem with a close friend, neighbour or relative;
- Being made redundant or sacked from your job;
- Looking for work without success for >1 month;
- Major financial crisis, like losing an equivalent of 3 months income;
- Problem with police involving court appearance;
- Something you valued being lost or stolen;
- Bullying;
- Violence at work;
- Violence at home;
- Sexual abuse;
- Being expelled from school;
- Running away from your home;
- Being homeless.

The list was developed as part of the British Psychiatric Morbidity Survey Programme [34, 46], and has previously been used in Tanzania and Kenya [17, 40]. We used the same list for comparability with these studies. Scores were grouped into “none”, “one”, “two” and “three or more” life events.

Perceived lack of social support was assessed from respondents’ answers to seven questions which were used in the 1992 Health Survey for England [47], and the British Surveys of Psychiatric Morbidity [34–36]. The seven questions take the form of statements that individuals could say were not true, partly true or certainly true for them in response to the question ‘There are people I know who: (i) Do things to make me happy; (ii) Who make me feel loved; (iii) Who can be relied on no matter what happens; (iv) Who would see that I am taken care of if I needed to be; (v) Who accept me just as I am; (vi) Who make me feel an important part of their lives; and (vii) Who give me support and encouragement’. Results were categorised into no, moderate or severe lack of perceived social support.

Social network size was assessed by respondents’ answers to three questions which have also been used in the British Surveys of Psychiatric Morbidity, namely:

- How many adults who live with you do you feel close to?
- How many relatives aged 16 or over who do not live with you do you feel close to?
- How many friends or acquaintances who do not live with you would you describe as close or good friends?

Responses to each of the three questions were added arithmetically into a total social network score.

Specific questions were also asked about caring responsibilities (Do you give care due to long term physical or mental disorder or disability? If yes, how much time do you spend giving care in a week?); about growing up with one natural parent or two until age 16; and about spending time in an institution before the age of 16.

### Statistical analysis

We examined the prevalence of suicidal ideation and suicide attempts, the prevalence of CMD, non-psychotic symptoms, alcohol consumption, hazardous drinking and substance abuse, and the predictors of suicidal thoughts and attempts, using STATA [48] to calculate unadjusted and adjusted odds ratios. Households were categorized into different socio-economic levels using an index of household assets. The index was constructed applying the principal component analysis procedure, as a proxy indicator for socio-economic status. Type of house, roofing and walling material, source of water, toilet facility and land ownership were included in the asset index [49, 50].

### Ethics

Ethical approval was granted by the KCL and KEMRI boards of research ethics respectively, and permission was obtained to conduct the study in households in Maseno division, which is part of the KEMRI/WRP Kombewa HDSS. Written and witnessed informed consent was sought from participants to take part in the study.

## Results

Out of the 1190 selected households, 1,158 participants consented to the study while 32 refused to participate in the study interviews, giving a response rate of 97.3 %.

Table 1 shows the prevalence of different stages of suicidal ideation and the prevalence of suicidal attempts. A quarter of the participants (24.1 %) had thought that life was not worth living (tedium vitae) at some point in their lives, while a fifth had experienced death wishes at some stage. 7.9 % reported suicidal thoughts and 1.9 % had made actual suicide attempts at some point in their lives.

**Table 1:** Prevalence of suicidal ideation and suicide attempts in last week, last year, at some other time and lifetime

	Last week: n (%) [95 % C.I]	Last year: n (%) [95 % C.I]	At some other time: n (%) [95 % C.I]	Life time: n (%) [95 % C.I]
Ever felt life not worth living	40 (3.5) [2.5 to 4.8]	175 (15.5) [13.4 to 17.7]	98 (8.7) [7.1 to 10.4]	273 (24.1) [21.6 to 26.7]
Ever wished were dead	34 (3.0) [2.1 to 4.2]	152 (13.4) [11.5 to 15.5]	65 (5.7) [4.5 to 7.3]	217 (19.2) [16.9 to 21.6]
Ever thought of taking life: Suicidal thoughts	8 (0.7) [0.3 to 1.4]	47 (4.2) [3.1 to 5.5]	42 (3.7) [2.7 to 5.0]	89 (7.9) [6.4 to 9.6]
Ever made attempt to take life: Suicidal attempts	6 (0.5) [0.2 to 1.1]	13 (1.2) [0.6 to 2.0]	8 (0.7) [0.3 to 1.4]	21 (1.9) [1.2 to 2.8]

The prevalence of suicidal thoughts was 0.7 %, 4.2 %, 3.7 % and 7.9 % for last week, last year, at some other time, and lifetime respectively, while the prevalence of suicidal attempts was 0.5 %, 1.2 %, 0.7 and 1.9 % respectively.

Numbers were too low to do multivariate analysis on suicidality in the last week and in the last year, so we only conducted multivariate analysis on life time suicidal thoughts and life time suicidal attempts. Table 2 shows the unadjusted risk factors for life time suicidal thoughts.

**Table 2:** Prevalence of lifetime suicide thoughts and their relationship with sociodemographic and psychosocial variables using univariate analysis (unadjusted odds ratios)

Factors		N	Prevalence: n (%)	Unadjusted OR (95 % C.I)	p-value
Lifetime suicide thought		1133	89 (7.9)		
Sex	Male	597	31 (5.2)	1	-
	Female	536	58 (10.8)	2.2 (1.41 to 3.48)	0.001
Age category	<30 years	278	26 (9.4)	1	-
	30-60 yrs	443	33 (7.5)	0.8 (0.46 to 1.34)	0.365
	>60 yrs	170	14 (8.2)	0.9 (0.44 to 1.72)	0.688
Household size	<=6 people	564	39 (6.9)	1	-
	>6 people	569	50 (8.8)	1.3 (0.84 to 2.01)	0.243
Marital status	Married/cohabiting	707	51 (7.2)	1	-
	Single	181	14 (7.7)	1.1 (0.58 to 2.00)	0.810
	Widowed/divorced	244	24 (9.8)	1.2 (0.84 to 2.33)	0.192
Education level	None	128	12 (9.4)	1	-
	Primary	616	51 (8.3)	0.9 (0.45 to 1.69)	0.686
	Secondary	320	22 (6.9)	0.7 (0.34 to 1.49)	0.588
	Post secondary	69	4 (5.8)	0.6 (0.18 to 1.92)	0.385
Employment status	Unemployed	559	37 (6.6)	1	-
	Self employed	476	45 (9.5)	1.5 (0.94 to 2.32)	0.094
	Employed	98	7 (7.1)	1.1 (0.47 to 2.51)	0.848
Immunity	≥500 cells/μL	388	15 (3.9)	1	-
	<500 cells/μL	61	4 (6.6)	1.7 (0.56 to 5.44)	0.337
Any CMD	No	1019	67 (6.6)	1	-
	Yes	114	22 (19.3)	3.4 (2.01 to 5.76)	<0.001
Malaria	Negative	687	51 (7.4)	1	-
	Positive	263	18 (6.8)	0.9 (0.52 to 1.60)	0.752
Asset Groups	Highest, Q1	400	25 (6.3)	1	-
	Q2	404	33 (8.2)	1.3 (0.78 to 2.29)	0.295
	Lowest, Q3	329	31 (9.4)	1.6 (0.90 to 2.70)	0.112
Perceived lack of support	No lack: 0	3	1 (33.3)	1	-
	Moderate lack: 1-7	311	26 (8.4)	0.2 (0.02 to 2.08)	0.171
	Severe lack: 8+	816	62 (7.6)	0.2 (0.01 to 1.84)	0.143
Total life events	0-1	353	14 (4.0)	1	-
	2-3	472	41 (8.7)	2.3 (1.24 to 4.30)	0.009
	4 or more	308	34 (11.0)	3.0 (1.58 to 5.71)	0.001
Total group size	3 or less	143	2 (1.4)	1	-
	4 to 8	513	41 (8.0)	6.1 (1.46 to 25.63)	0.013
	9 or more	474	46 (9.7)	7.6 (1.82 to 31.61)	0.005
Being a carer for more than 4 hours a week	No	24	1 (4.2)	1	-
	Yes	170	14 (8.2)	2.1 (0.26 to 16.45)	0.494
Spent time in an institution before age 16	No	906	73 (8.1)	1	-
	Yes	220	16 (7.3)	0.9 (0.51 to 1.57)	0.699
Did not have both natural parents at home until age 16	No	954	77 (8.1)	1	-
	Yes	172	12 (7.0)	0.9 (0.45 to 1.61)	0.625
Any Psychotic Symptoms	No	953	66 (6.9)	1	-
	Yes	155	21 (13.6)	2.1 (1.25 to 3.56)	0.005

Factors associated with life time suicidal thoughts at the bivariate level included being female, having CMD, experiencing recent life events, having a large social network size and having psychotic symptoms.

Table 3 shows the adjusted odds ratios for risk factors for life time suicidal thoughts.

**Table 3:** Relationship of lifetime suicidal thoughts with factors significant at the bivariate level, using odds ratios adjusted for each other by logistic regression analysis

Factors		Adjusted OR* (95 % C.I)	p-value
Sex	Female	1.8 (1.11 to 2.90)	0.017
Any CMD		2.7 (1.49 to 4.89)	0.001
Total life events	2-3	2.3 (1.23 to 4.42)	0.001
	4 or more	2.6 (1.35 to 5.06)	0.004
Total support group	4 to 8	7.7 (1.82 to 32.88)	0.006
	9 or more	9.1 (2.15 to 38.49)	0.003
Any Psychotic symptoms		1.4 (0.79 to 2.43)	0.252

Factors associated with life time suicidal thoughts at the multivariate level include being female, having CMD, having a number of recent life events and having a large social group size which all remained significantly associated with increased rates of suicidal thoughts after adjustment for the other variables significant at the bivariate level but psychotic symptoms were no longer significant risk factors after adjustment for the other variables.

Table 4 shows the prevalence of life time suicidal attempts and and their relationship with sociodemographic and psychosocial variables using univariate analysis (unadjusted odds ratios).

**Table 4:** Prevalence of life time suicidal attempts and and their relationship with sociodemographic and psychosocial variables using bivariate analysis (unadjusted odds ratios)

Factors		N	Prevalence: n (%)	Unadjusted OR (95 % C.I)	p-value
Lifetime Suicide attempt		1133	21 (1.9)		
Sex	Male	597	11 (1.8)	1	-
	Female	536	10 (1.9)	1.0 (0.43 to 2.40)	0.977
Age category	<30 years	278	8 (2.9)	1	-
	30-60 yrs	443	7 (1.6)	0.5 (0.19 to 1.51)	0.242
	>60 yrs	170	2 (1.2)	0.4 (0.08 to 1.91)	0.252
Household size	<=6 people	564	6 (1.1)	1	-
	>6 people	569	15 (2.6)	2.5 (0.97 to 6.54)	0.058
Marital status	Married/cohabiting	707	12 (1.7)	1	-
	Single	181	4 (2.2)	1.3 (0.42 to 4.11)	0.645
	Widowed/divorced	244	5 (2.1)	1.2 (0.42 to 3.47)	0.721
Education level	None	128	2 (1.6)	1	-
	Primary	616	11 (1.8)	1.1 (0.25 to 5.23)	0.861
	Secondary	320	8 (2.5)	0.6 (0.34 to 7.71)	0.548
	Post-secondary	0	-	-	-
Employment status	Unemployed	559	6 (1.1)	1	-
	Self employed	476	14 (2.9)	2.8 (1.06 to 7.33)	0.037
	Employed	98	1 (1.0)	1.0 (0.11 to 7.98)	0.962
Immunity	≥500 cells/μL	388	8 (2.1)	-	-
	<500 cells/μL	61	0	-	-
Any CMD	No	1019	17 (1.7)	1	-
	Yes	114	4 (3.5)	2.1 (0.71 to 6.48)	0.177
Malaria	Negative	687	11 (1.6)	1	-
	Positive	263	6 (2.3)	1.4 (0.53 to 3.91)	0.481
Asset Groups	Highest, Q1	400	4 (1.0)	1	-
	Q2	404	12 (3.0)	3.0 (0.97 to 9.48)	0.057
	Lowest, Q3	329	8 (2.4)	1.5 (0.41 to 5.74)	0.530
Perceived lack of support	No lack: 0	3	0	-	-
	Moderate lack: 1-7	311	6 (1.9)	1.1 (0.40 to 2.73)	0.920
	Severe lack: 8+	816	15 (1.8)	-	-
Total life events	0-1	353	2 (0.6)	1	-
	2-3	472	14 (3.0)	5.4 (1.21 to 23.76)	0.027
	4 or more	308	5 (1.6)	2.9 (0.56 to 15.03)	0.206
Total group size	3 or less	143	3 (2.1)	1	-
	4 to 8	513	8 (1.6)	0.7 (0.19 to 2.82)	0.659
	9 or more	474	10 (2.1)	1.1 (0.27 to 3.71)	0.993
Being a carer for more than 4 hours a week	No	24	0	-	-
	Yes	170	5 (2.9)	-	-
Spent time in an institution before age 16	No	906	18 (2.0)	1	-
	Yes	220	3 (1.4)	0.7 (0.20 to 2.34)	0.542
Did not have both natural parents at home until age 16	No	954	18 (1.9)	1	-
	Yes	172	3 (1.7)	0.9 (0.27 to 3.17)	0.899
Any Psychotic Symptoms	No	953	10 (1.1)	1	-
	Yes	155	9 (5.8)	5.8 (2.32 to 14.55)	<0.001

Factors associated with suicidal attempts at the bivariate level include being self-employed, experiencing 2–3 life events and having psychotic symptoms.

Table 5 shows the adjusted odds ratios for risk factors for life time suicidal attempts.

**Table 5:** Relationship of lifetime suicidal attempts with factors significant at the bivariate level, using odds ratios adjusted for each other by logistic regression analysis

Factors		Adjusted OR* (95 % C.I)	p-value
Employment status	Self employed	2.3 (0.87 to 6.31)	0.093
Total life events	2-3	3.0 (0.64 to 13.88)	0.164
	4 or more	2.0 (0.38 to 10.89)	0.406
Any Psychotic symptoms		5.1 (2.00 to 13.03)	0.001

Having any psychotic symptoms was the only factor associated with suicide attempts after adjustment for other factors significant at the bivariate level.

## Discussion

### Overall findings

This analysis of suicidal thoughts and attempts derived from a mental health epidemiological survey of a household population in Maseno district, Nyanza Province in Kenya, found that over 25 % of the participants had thought that life was not worth living (tiredness of life or tedium vitae) at some point in their lives, while over 20 % had experienced death wishes at some stage. One twelfth reported suicidal thoughts and around one fiftieth had made actual suicide attempts at some point in their lives. In the adjusted analysis of factors associated with suicidal thoughts, being female, having CMD, having a number of recent life events and having a large social group size were all associated with increased rates of life time suicidal thoughts, but psychotic symptoms were no longer significant after adjustment for the other variables. In the adjusted analysis of suicide attempts, having any psychotic symptoms was the only factor associated with suicide attempts after adjustment for other factors significant at the bivariate level.

Previous studies of suicidal ideation in Kenya have focussed on samples of psychiatric inpatients [51] and outpatients [52], general medical inpatients and outpatients [53], primary care [54] and college students [55], but there have been no household population studies before this one. A comparison between students from four African countries and the US found that the prevalence of suicidal ideation ranged between 23.1 % and 31.9 % in the four African countries and was 16.9 % in the USA [56].

The World Health Organization World Mental Health Survey Initiative which studied 17 countries (including Nigeria and South Africa but not Kenya) found that the cross national prevalence of suicidal ideation and attempts was 9.2 % and 2.7 % respectively which is broadly similar to our findings in Kenya [57]. The national comorbidity survey in the US found prevalence rates of 2.8 % and 3.3 % suicidal thoughts and 0.7 % and 0.8 % suicidal attempts (and suicidal gestures combined), in the surveys of 1990–2 and 2001–3 respectively [8]. A national household survey of adults in Britain [12], found that a fifth of the sample [20.3 %] had thought that life was not worth living (tedium vitae) at some point in their lives, while death wishes and suicidal thinking were reported by a sixth of the sample, and 4.4 % made suicide attempts. A national survey in Australia found a life time risk of 16 % for suicidal thoughts and 3.6 % for suicide attempts [58]. Thus we found that suicidal ideation in this Kenyan population is more common than suicidal ideation in the UK and Australia, while suicidal attempts in Kenya were more common than that found in the US but rare than found in the UK. The only recent US survey reporting prevalence of desire for death is that by Baca Garcia [13] which found lifetime prevalence of 11.3 % and 10.2 % in two large US surveys compared to 19.2 % found in our Kenya study.

Risk factors associated with suicidal thoughts and attempts show some consistency across studies and countries. The World Mental Health Survey found that risk factors for suicidal ideation which were consistent across the 17 countries included being female, younger age, less well educated, unmarried (single, separated, divorced or widowed) and having a mental disorder [57]. The US National Comorbidity Survey found that higher rates were associated with being female, young age, less well educated, unmarried (single, separated, widowed and divorced) and unemployed [8]. An analysis of suicidal ideation and suicide attempts among Hispanic Subgroups in the US found that 45–64 year olds were at high risk [59, 60].

Since many studies (eg [12, 57, 59, 60]) have found an association between suicidality and age, it is surprising that an association between age and either suicidal ideation or attempts was not found in this study. A detailed cohort study in the UK study found that the decrease in reported previous-year suicidal thoughts with increasing age [59, 60] was partly explained by lower rates of reported abuse in childhood (in those aged 75+), depression, and anxiety symptoms (in those aged 55+), factors which are all strongly associated with suicidal thoughts; and partly by higher rates of protective factors in people aged 35+, specifically homeownership and cohabitation. Rates of phobias, irritability and compulsions also decreased with age, and the association of these symptoms with suicidal thoughts was particularly strong in the youngest (16–34) age group. People who reported experiencing childhood abuse in all age groups reported more suicidal thoughts, suggesting abuse has lifelong negative effects on suicidal ideation (59). It is possible that in our Kenyan sample, the older age groups have accumulated less protective factors than would be found in a Western sample.

The relationships we found between life events and suicidal ideation and attempts respectively were to be expected, and are found elsewhere [61] but the inverse relationship of suicide ideation and attempts with social network size is counter-intuitive. However, in the same survey we have found a similar inverse relationship of CMD and psychosis with social network size and perceived social support (unpublished papers). Similarly a previous study in Tanzania found a relationship of CMD with three or more recent life events but no relationship with social network size or perceived social support [40]; and a relationship of psychosis with two or more recent life events, but again no relationship with social network size of perceived social support [44]. Disturbed interpersonal relationships are an important precipitant of suicidal behaviour in the African setting, especially in young females [62]. It could be that those with a large network of friends are likely to suffer more shame and guilt if they have adverse life events, for example, childlessness and divorce which the woman may perceive as her own fault, hence leading to suicidal thoughts and self-harm. The disadvantaged position that women often occupy may contribute to the risk of suicidal ideation and attempts.

The relationships we found in the adjusted analysis between CMD and lifetime suicidal ideation, and between psychotic symptoms and suicide attempts are to be expected and are found in the world mental health survey in 17 countries [57] and in the UK [63, 64]. However, the multivariate analysis did not demonstrate significant associations between psychotic symptoms and suicidal ideation, and between CMD and life time suicidal attempts.

### Strengths of study

The strengths of the study are the use of a health and demographic surveillance site for the random sample of households, the high response rate, and the systematic approach to the clinical and socio-demographic assessments. The population in the surveillance site is regularly monitored by field staff who visit each household bi-annually to capture health and demographic information (birth rates, death rates, causes of death, pregnancies, immunization status, in- and out-migrations, etc.). Various studies nested on the HDSS platform take advantage of the sampling frame inherent in the HDSS, whether at individual, household/compound or regional levels. This familiarity with survey procedures is likely to have been influential in the achievement of a high response rate.

### Limitations of study

The findings of the study are limited by the fact that the cross-sectional design of the study does not allow causality to be inferred in the risk factor associations. It is also important to note that all the data was collected by a standardised structured research interview and was not confirmed by an interview with an expert psychiatrist, or by clinical records. People may be less willing to confide suicidal thoughts to a research interviewer than to an expert psychiatrist in a clinical situation.

The fact that the question about attempted suicide was only asked of those who had suicidal ideation might have left out the impulsive suicide attempts that are not preceded by suicidal ideation. This might have led to an underestimation of the true rate of attempted suicide. Furthermore, the question about attempted suicide gives only one example of a method, namely overdose of tablets, to help the participant understand what is meant by a suicide attempt. It is possible that not giving other examples may have led to under-reporting of suicide attempts by other methods, but since the question also says “or in some other way?”, we think it unlikely that participants would not recall suicide attempts which used other methods such as pesticides or asphyxiation.

Our data was collected via interview, which was then input to the PDA by the interviewer, rather than collected via self-completion and put directly onto the PDA by the respondent. Studies in the West show that higher rates are reported by self-completion than by interviewer-completion (e.g., [65]). Suicidality is regarded as a sin in religious cultures and this would increase discomfort in revealing suicidality in an interview, even to a health worker. Our suicide questions and other instruments were all reviewed by a local expert Luo psychiatrist and considered culturally relevant to the context.

The implementation of the study was hampered by a number of logistical challenges including the difficult terrain which posed problems for local transport for research staff and continuing administrative difficulties which led to delays in the implementation of the project. The interviewing period, initially planned to last 3 months, took place over a period of 6 months and was temporarily halted for several weeks over the period of the 2013 election due to further fears of election unrest.

## Conclusion

This study indicates that suicidal ideation and attempts pose a significant public health burden in this poor rural area of Kenya, characterised by political unrest, high unemployment and environmental problems of drought, and water hyacinth in the Lake hampering the fishing industry. This is a local rather than a national survey and there is a need for a nationally representative mental health survey in Kenya which includes an appraisal of suicidal ideation and attempts.

The findings are relevant for mental health promotion and prevention programmes, public education and professional training programmes in relevant sectors, especially in front line health workers and social workers who need regular systematic training in biopsychosocial assessment and management of suicidal risk.

## Abbreviations

AUDIT: Alcohol use disorder identification test
CIS-R: Clinical interview schedule-revised
CMD: Common mental disorder
HDDS: Health and demographic surveillance system
KEMRI: Kenya medical research institute
KMTC: Kenya medical training college
ONS: Office of national statistics, UK
PDA: Personal digital assistant (electronic handheld information device)
WRP: Walter reed project
